# Pictorial spacecrafts – the Ames’ Glass

**DOI:** 10.1177/20416695251396335

**Published:** 2025-12-22

**Authors:** Maarten W. A. Wijntjes, Lianne M. E. Pinkse

**Affiliations:** 1 Perceptual Intelligence Lab, 578936 Delft University of Technology, Delft, the Netherlands

**Keywords:** 3D perception, depth, stereopsis, binocular vision

## Abstract

There are various ways to evoke stereopsis without binocular disparities. Closing one eye, or looking through a synopter are well-known methods. Ames (1925) listed nine ways of generating this so-called ‘‘plastic effect,” one of which involves a cylindrically curved lens placed in front of one eye. We investigated qualitative perceptual effects of this particular way of viewing artworks. A total of 38 participants viewed three digitally reproduced paintings. Initially, they were asked to spontaneously report the perceptual effect of the lens. While being naive to the purpose of the experiment, 66% of the participants reported increased depth experience. In addition, participants reported increased contrast, color vibrancy, and material expression (e.g., increased shininess). During a second part of the experiment, we asked to report on seven qualities: depth, color, three-dimensional shape, realism, detail, light, and material. All qualities increased significantly except detail, which seemed to show idiosyncratic results: the majority of the observers experienced a decrease of detail, while a minority reported, surprisingly, an increase of detail. The results agree with previous qualitative accounts on monocular aperture viewing, despite relying on entirely different nonpictorial cues: monocular aperture viewing relies on the absence of vergence and binocular disparities, whereas the Ames’ Glass relies on distorted binocular disparities while keeping vergence unchanged. Together with the synopter, for which qualitative data is lacking, the Ames Glass and monocular aperture viewing are pictorial spacecrafts fit for art gallery viewing.

## How to cite this article

Wijntjes, M. W. A., & Pinkse, L. M. E. (2025). Pictorial spacecrafts – the Ames' Glass. *i-Perception*, *16*(6), 1–16. https://doi.org/10.1177/20416695251396335

## Introduction

In 1838, Wheatstone (1838) invented the stereoscope (Wade, 2012): a device that delivers different pictures to both eyes. If these pictures are taken from two slightly different vantage points (corresponding to the eye positions), this results in a vivid three-dimensional (3D) experience. Since the invention, the technique has been used in various popular applications such as handheld stereoscopes, 3D movies and VR masks. The difference between the two images (binocular disparities) correspond to differences in relative depth which is processed in the primary striate cortex and used for depth discrimination (Barlow et al., 1967). Surprisingly, a comparably vivid impression of three-dimensionality can be experienced in the absence of binocular disparities. The effect is sometimes (somewhat confusingly) referred to as ‘‘monocular stereopsis’’ (Howard & Rogers, 2012) because one way to induce it is to close one eye. Binocular and monocular stereopsis share a phenomenological resemblance (Vishwanath, 2014), but also see Linton (2017) for an alternative view.

**Figure 1.**
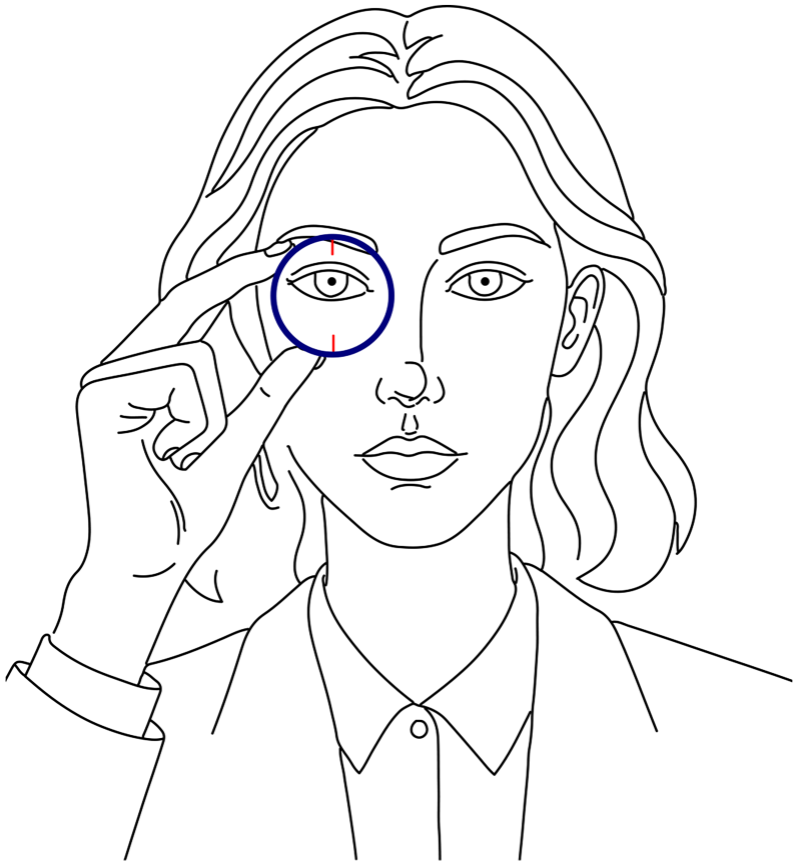
Illustration of the use of the Ames’ Glass, a cylindrical lens in front of one eye, with the axis vertical as indicated by the red lines. Crucially, the other eye is also open.

Exactly 100 years ago, Ames (1925) published nine ways of inducing this stereoscopic impression when viewing two-dimensional (2D) pictures. The first method was simply closing one eye, referring to one of the earliest sources describing the experience of monocular stereopsis (Claparède, 1904; O’Shea, 2017). The list continues with various methods that have been commented on previously (Blundell, 2015; Schlosberg, 1941; Wijntjes et al., 2016) although many have never been thoroughly tested. One method concerned the use of a cylindrically curved lens held in front of one eye, while keeping the other eye open (Figure 1):

*Looking at a picture binocularly, with one eye receiving a sharp image and the other a blurred one*.—A blurred image can be produced either by a positive or negative lens or cylinder before one eye. The best effect is produced by a plus cylinder with its axis vertical. At normal reading distance a +3 to +4D cylinder is good. This blurs the image in the horizontal direction. When the picture is viewed binocularly this blurring is at least partially suppressed and we see the picture sharp and clearly with a marked illusion of depth much more than we get from one eye alone. Why we get a greater illusion than with one eye is hard to explain. The fact that a better effect is obtained with a cylinder, which diffuses all vertical lines and leaves the horizontal ones sharp, than with a spherical lens which gives a general diffusion suggests that our stereoscopic binocular processes are in some way utilized. Possibly there is a suppression of all parts of the blurred images except that part which with the clear image in the other eye forms a binocular stereoscopic combination in conformity with depth suggestions given us by other faculties.

In this study, we aim to empirically investigate Ames’ claim concerning this cylindrical lens. Before doing so, we will briefly review the subject of stereoscopic impression from 2D images.

### Phenomenology of Stereopsis

Stereopsis literally means solid (stereo) appearance (opsis) and is often used interchangeably with binocular stereopsis (Howard & Rogers, 2012). Here, the addition ‘‘binocular’’ suggests the source of depth information: two slightly different perspectives leading to the so-called binocular disparities. Monocular stereopsis is the increase of pictorial depth when closing one eye. Studies on monocular stereopsis have been both qualitative and quantitative in nature. Initial accounts, such as Claparède (1904), note the separation between foreground and background “favored by the sharpness of the outlines, and by the play of shadow and light” (O’Shea, 2017). A few years before, Ebbinghaus (1902) had already noted that looking at a painting with one eye made ”the water surfaces, avenues, columned halls visibly extend away from the viewer from bottom to top, as they are painted; the illusion comes much closer to the impression of physical reality than with binocular viewing.”

The first quantitative account on this depth perception came from Koenderink et al. (1994) investigating the synopter (von Rohr, 1907): a hand held viewing device that creates identical perspectives in both eyes by using mirrors and half-mirrors. Thus, the synopter nullifies binocular disparities. Using a depth probe task, Koenderink et al. (1992) let observers look binocularly, monocularly and synoptic to a depicted 3D relief and found that the pictorial relief viewed through the synopter evoked the most pronounced sense of depth. In a later study, using a similar experimental paradigm, Koenderink et al. (2013) found comparable results for a different optical device: the zograscope^1^ (discussed in the following section).

While using a different experimental paradigm, Vishwanath and Hibbard (2013) could not reproduce this quantitative effect, but did find a variety of qualitative effects associated with monocular stereopsis. Also, they found considerable individual differences. For example, when they initially screened 31 participants by letting them report differences between viewing a real, 3D object (a potted flowering plant), 22 (i.e., 71%) reported a difference between monocular and binocular viewing. When letting observers view pictures with one eye, through an aperture, their verbal descriptions resembled those reported in a peculiar opposite case of Barry (2009) who, after recovering from strabismus and eye jitter could suddenly experience binocular stereopsis at the later age. These qualifications included a higher sense of realism, clearer depth separation, more three-dimensional, closer, better color, shinier, clearer texture, deeper shadows, material characteristics, and more.

### Generating Stereopsis

The account of stereopsis by Ebbinghaus (1902) actually not only concerned monocular viewing, but particularly looking monocularly through a ‘‘hohle hand,” that is, cupped hand. This addition of an aperture served as an inspiration for the optical device ‘‘Ein Einfaches Plastoskop” (a simple plastoscope) described by Zoth (1916). The viewing aid was not more than a slightly converging cylinder with a rectangular aperture at the end. Despite (or perhaps because of) its simplicity, the device signaled a desire to engage with tangible tools rather than merely closing one eye. A more complex, and at the time patented optical device was the synopter (von Rohr, 1907) which optically places the eyes at the same vantage point by the use of half and full mirrors. This single vantage point certainly also induced stereopsis (Wijntjes et al., 2016) and moreover passed a placebo test (Wijntjes, 2017). The original synopter never made it into production, at least not to our knowledge. Since the synopter resurfaced through (Koenderink et al. 1994), it has been produced sporadically. Interestingly, long before this time, Victorian optical devices to view pictures had been popular and widely produced. The two most popular devices were the zograscope and graphoscope. They both rely on a large (bi)convex lens that covers both eyes (Koenderink et al., 2013). As explained by Michaud (1908), the lens effectively works as two prisms at the location of the eyes and induces parallel vergence (eyes oriented towards a point far away). Other optical side effects that may not have been intended but are present are chromostereopsis, and the eye lens accommodation is (ideally) at infinity. An additional effect induced by the zograscope may be caused by the mirror acting as an aperture, which has been shown to induce stereopsis (Da Vinci, 1888; Higashiyama & Shimono, 2012). A lesser known device is the Megalethoscope (Bantjes, 2021) which resembles a graphoscope but uses additional light sources, including from the back.

Until now we have not yet touched upon what actually causes the effect. It is clear that in all cases, the nonpictorial depth cues like accommodation, vergence and binocular disparities, are impaired. This means that the signal of the physical picture flatness is impaired, which could release the pictorial cues from their flatness captivity. In Table 1, we present an overview of devices known to generate stereopsis from 2D pictures. The account of some sort of cue combination between pictorial and nonpictorial cues can be found throughout the literature (Ames, 1925; Claparède, 1904; Koenderink et al., 1994; Schlosberg, 1941). However, Vishwanath (2014) proposed a different theory: in the absence of binocular disparities and other information signaling the pictures’ flatness (like the edge, which is made invisible by using an aperture), the brain attributes accommodation to the pictorial scene. When the brain attributes a nonpictorial cue to the sum of the pictorial cues, that is, to the representation instead of the physical medium, the experience becomes what one could call ‘‘nonpictorial.” As the accommodation signal is at the distance of the picture, it would be expected that the scene appears miniaturized and closer, which is indeed what was found (Vishwanath & Hibbard, 2013).

**Table 1.** Overview of pictorial space crafts. Also consult Wijntjes et al. (2016) for a pictorial overview.

Device	Frame	Accommodation	Vergence	Disparities	Distance
Zograscope	Yes	Far	Parallel	Far/chromo	Fixed
Graphoscope	No	Far	Parallel	Far/chromo	Fixed
Perspectoscope	No	Far	Parallel	Far	Fixed
Shomescope	No	Far	Parallel	Far	Fixed
Verant	No	Far	n.a.	No	Fixed
Plastoscope	Yes	Normal	n.a.	No	Variable
Synopter	Yes	Normal	Parallel	Far	Variable
Ames’ Glass	No	Normal ${}^{\text{a}}$	Normal ${}^{\text{b}}$	Disturbed	Variable

${}^{\text{a}}$ It is not entirely clear how the cylindrical lens affects accommodation.

${}^{\text{b}}$ Likely vergence is somewhat affected but not ‘‘parallel’’ as in the other cases.

### The Ames’ Glass

The list of Ames (1925) does not specifically refer to existing viewing devices like those discussed in the previous section. There is mention of Javal’s Iconoscope, that functionally resembles a synopter (but still maintains a minute difference in binocular vantage points). Yet, there is little other reference to viewing aids, particularly those designed to elevate pictorial experiences. As there is a history of art, there is also a history of viewing, which is related to the history of viewing devices. Optical aids such as the graphoscope, zograscope, synopter, snapscope, shomescope, perspectoscope, plastoscope, and megalethoscope have all been designed (and except for the synopter, been produced) to elevate pictorial experience. Although there is no history yet, we situate the Ames’ Glass in this tradition, emphasizing its relation to pictorial experience. As an umbrella category for these devices we suggest Pictorial Space Crafts.

**Figure 2.**
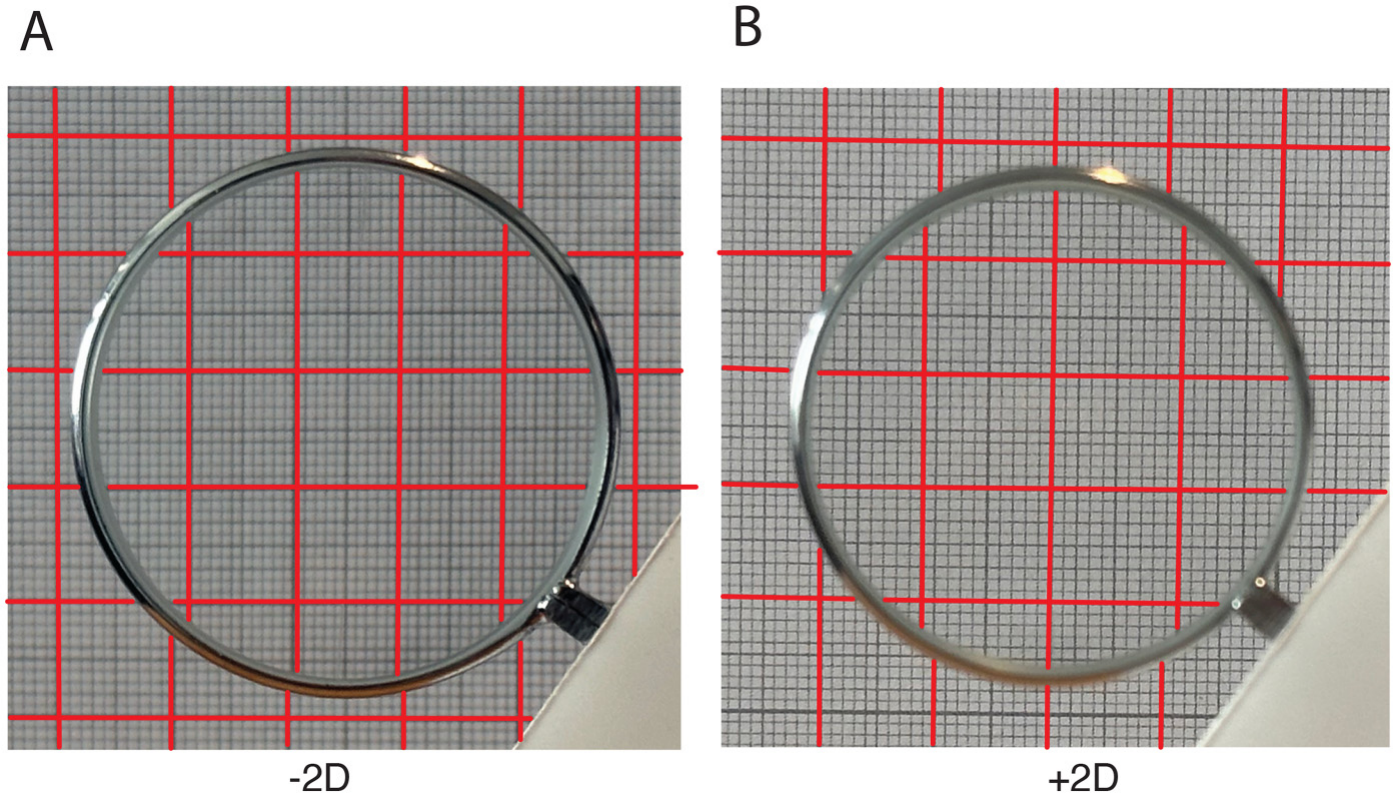
The optical distortions of two ±2 D lenses used in the experiments. As can be seen: only the vertical lines are displaced because the curvature is in the horizontal direction. The grid lines have been emphasized by the red lines for clarity, but the actual background can be seen when zoomed in (A). The defocus blur in (B) was accidental and unrelated to the lens itself.

The Ames’ Glass is a cylindrically curved lens, with the curvature in the horizontal direction, held in front of one eye with both eyes open (see Figure 1). It is difficult to exactly predict the effect of an optical device as it is unknown how the eye itself reacts, for example by accommodation Koenderink et al. (2013, p. 195). To at least have some optical characterization of the cylindrical lens, we photographed a millimeter grid through the positive and negative cylindrically curved lens (Figure 2). It can be clearly seen that the negative lens compresses the grid horizontally, while the positive lens expands the grid horizontally. Also note that there is no vertical distortion (as the horizontal lines are continuous). It is not known how accommodation nor vergence would react and, therefore, it is difficult to predict the optical effect. If the accommodation of the lens stays unchanged (with respect to the eye without the lens), the retinal image may become de-focused resulting in horizontal blur “diffusing vertical lines” (see quote in introduction). This impairs the detection of the (horizontal) disparities and thus affects depth perception. The binocular rivalry resulting from presenting a sharp and blurred image in either eye is mitigated by the tendency of sharp images being perceptually dominant over a blurred image (Arnold et al., 2007).

There is not much empirical evidence for the effect of the Ames’ Glass. The only indirect evidence comes from Wijntjes (2017) who investigated a spherical lens of +3 diopters. They found stereopsis was higher than for a placebo device, but weaker than for the synopter. The current study differs from this earlier study in three substantial ways. Firstly, Wijntjes (2017) only asked observers about their overall impression without investigating the qualitative dimensions characterizing stereopsis (Vishwanath & Hibbard, 2013). Secondly, the lens was spherical instead of cylindrical. Thirdly, the strength of the lens was +3 diopters, in line with Ames (1925), but in the current study we will explore weaker strengths. The heavy blur introduced by a +3D spherical lens is rather distracting and seems to affect stereopsis negatively. When we informally tried a weaker, cylindrical lens instead, and particularly with negative instead of positive cylindrical curvature, stereopsis seemed much more pronounced. The motivation of the current study is to confirm the effect of the Ames’ Glass by investigating its qualitative dimensions, which can be compared to those found in monocular stereopsis (Vishwanath & Hibbard, 2013). Moreover, we were interested in what strength and polarity (plus or minus) observers would naturally prefer.

## Method

### Participants

A total of 38 volunteers participated in the experiment, almost all were students at the faculty of Industrial Design Engineering of the TU Delft. The average (standard deviation) age was 23.8 ( ±2.0 ). All participants had normal ( N= 26) or corrected-to-normal ( N= 12) vision. The TNO test for stereoscopic vision (Walraven & Janzen, 1993) was used to measure stereo acuity. Two participants showed an acuity of 15 arc sec, 15 showed an acuity of 30 arc sec, 18 an acuity of 60 arc sec, two an acuity of 120 arc sec, and for one participant the test was not conducted. Thus, the vast majority showed good stereo acuity. To assess eye-dominance, we let observers move both index fingertips towards a imaginary line between their eye and a fixation point a few meters away, with one hand further than the other. When opening and closing left and right eye subsequently, the participants reported which eye they used to align the index fingertips with the target. For one participant the test was inconclusive, 10 participants reported left dominant eye, and 27 participants reported right dominant eye.

After the first batch of 12 participants we received feedback that the glasses caused some discomfort. Wearing ‘‘wrongly’’ prescribed glasses, can indeed cause eyestrain and headache which often happens after a recent moderate change in optical prescription (Denniston & Murray, 2018). We decided to shorten the experiment from six to four paintings, and using only +1 and − 1 lenses and not any stronger. Also, we decided to remove the Pollock painting from Phase 2 (see the Procedure section). After this intervention we did not receive any mention of eyestrain or discomfort. Because the experimental design changed somewhat, we will refer to Group A as the first 12 participants, and Group B as the remaining 26 participants.

### Stimuli and Apparatus

**Figure 3.**
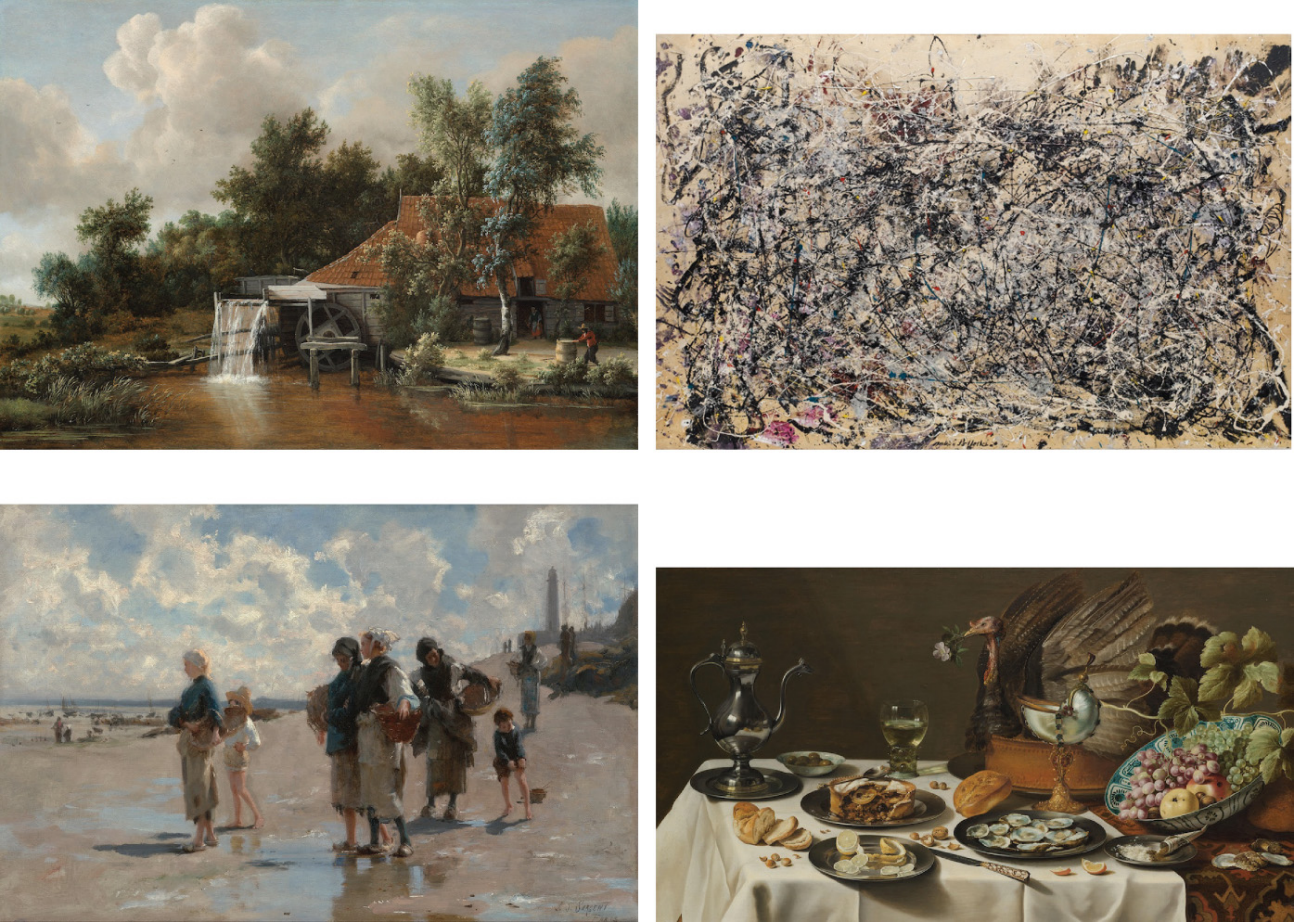
Stimuli from top left to bottom right: (1) Meindert Hobbema *A Watermill* (c. 1664) Rijksmuseum, (2) Jackson Pollock *Number 1A, 1948* (1948), © 2025 Digital image, The Museum of Modern Art, New York/Scala, Florence, (3) John Singer Sargent *Fishing for Oysters at Cancale* (1878) The Museum of Fine Arts Boston, and (4) Pieter Claesz *Still Life with a Turkey Pie* (1627) Rijksmuseum.

The four paintings that were used as stimuli are shown in Figure 3. The pictures were displayed on a $55^{\prime \prime }$ LG LCD screen with anti-glare coating that reduces reflections. Participants were seated behind an office table ∼ 3 m from the screen (the edge of the table was exactly 280 cm from the screen). As the screen was 121 cm wide, the horizontal viewing angle subtended 22.8 ${}^{\circ }$ .

We used lenses from a standard optometry trial lens set. Participants were instructed to hold the lens in front of one eye (details can be found in the Procedure section) while keeping the cylindrical lens orientation lines vertical. These small lines indicated the direction of zero curvature, which thus resulted in a horizontal distortion of the visual field.

### Procedure

The experimental procedure consisted of two phases. In the first phase, the participants were naive to the hypothesized effect of the lens in order to ensure spontaneous reactions. In the second phase, the experimenter specifically asked to address qualitative aspects associated with stereopsis. Although we did not specifically explain the hypothesized effects, these questions were suggestive and thus made the participants less naive.

#### Phase 1

A cylindrical lens of − 1D was given and the participants were instructed to hold it in front of their right eye ( N= 14) or left eye ( N= 24). Half of the participants held the lens in front of their dominant eye ( N= 19), 18 held the lens in front of their nondominant eye and of one participant eye dominance was not established. While keeping both eyes open (verified by the experimenter), the participants were asked to describe the effect of the lens (if any), specifically addressing the difference between viewing with and without the lens. The order of paintings was counterbalanced for different subgroups of participants.

At the end of Phase 1, participants were asked which painting yielded the strongest effect (whatever the effect may be). Viewing their preferred painting, they were given various other lenses between − 2D and +2D (for the first 12 participants, see Participant section for more information) and between − 1D and +1D (for the remaining participants) and asked which worked best. They were also asked to compare the effect between both eyes and choose the eye for which it worked best.

#### Phase 2

In the second phase, we asked specific questions about the effect and thereby somewhat gave away the purpose of the experiment and the hypothesized effect of the Ames’ Glass. Participants used their preferred lens on their preferred eye that they selected at the end of Phase 1. The task was to rate and comment the effect with respect to seven attributes using the following statements:

1. I sense a stronger sense of **depth** (planes).
2. The **colors** in the painting look more lively.
3. It felt more **three-dimensional** (curvature of objects).
4. The scene looked more **realistic**.
5. I could see more **details**.
6. The **shadows and light** are more salient.
7. **Material properties** looked more realistic.

### Data Analysis

Aside from rating and preference data, we also collected verbal responses during both phases of the experiment. To analyze these verbal responses we used Claude (Anthropic, 2025) model 3.7 Sonnet via the API. For the Phase 1 data, we used Claude to transform the responses in scale data to estimate whether observers perceived stereopsis. In Phase 2, we used Claude to create clusters from the responses on the seven specific questions.

## Results

### Phase 1: Spontaneous Remarks

Perceived stereopsis was initially assessed by the experimenter on the basis of the participants’ descriptions. The experimenter rated the effect using a Likert scale from 1 to 5. Although this score may not have any absolute value, it can be used to assess individual differences between observers related to stereo acuity and the interaction between eye dominance and lens position. As a second measure, we used Claude to quantify the effect on the basis of verbal responses. We first asked to summarize all responses per painting. From this, we extracted five features that emerged from this summary: (1) depth, (2) sharpness, (3) color vibrancy, (4) contrast, and (5) material. Then we asked Claude to rate all responses on the five above mentioned attributes between − 3 and 3, with 0 denoting no or an absent effect. In Table 2, the number of participants referring to a specific effect are presented.

**Table 2.** Types of stereoscopic effects experienced as spontaneously reported by the participants.

Characteristic	Positive Effect	No Effect	Negative Effect
Depth	25	13	0
Sharpness	5	8	25
Color vibrancy	9	28	1
Contrast	6	31	1
Material	9	22	9

To compare the ratings of the experimenter with the ratings of Claude, we correlated the experimenter impressions with the ratings by Claude on the Depth attribute. The Spearman rank correlation was highly significant ( ρ=0.77 , p<.0001 ) indicating strong similarity between the two variables. Neither variable is without shortcoming, the experimenter rating may be biased, while for Claude we do not know how reliable it performs. Given that Claude also (without knowing) extracted the typical stereopsis characteristics in the initial clustering analysis, we choose perceived stereopsis to be represented by the Claude ratings. In the remainder of this Phase 1 analysis, we used the Claude ratings as dependent variable to investigate the influence of lens characteristics and eye dominance.

We tested the relation between stereo acuity and perceived stereopsis (as quantified by the Claude ratings in depth). As the stereo acuity was not evenly distributed we did not perform a regression but rather compared two acuity values, $30^{\prime \prime }$ ( N=15 ) and $60^{\prime \prime }$ ( N=18 ). The average perceived stereopsis over the four paintings was 0.5 and 0.46, respectively, which was not significant according to a Mann-Whitney test ( U=157 , p>.05 ). Yet, it may be interesting to note that three out of 15 observers did not experience the effect in the most acute group ( $30^{\prime \prime }$ ), while seven out of 18 observers did not experience the effect in the less acute group ( $60^{\prime \prime }$ ).

Furthermore, we tested whether positioning the lens over the dominant eye affected perceived stereopsis. Eighteen participants had (in hindsight) the lens in front of their nondominant eye (mean effect = 0.48) and 19 participants in front of their dominant eye (mean effect = 0.51) which was not significant according to a Mann-Whitney test ( U=167 , p>.05 ). For one participant, we could not establish the dominant eye.

At the end of Phase 1, we tested participants’ preference for lens polarity (minus or plus) and eye (left or right) by letting them try out various configurations. Fifteen participants preferred the lens over their nondominant eye, 19 over their dominant eye and four (including the participant for who we did not establish eye dominance) did not have a preference. The perceived stereopsis (as quantified by the Claude analysis) was not statistically different between the nondominant and dominant group ( U=127 , p>.05 ). Preference for lens polarity was relatively equally distributed: 21 preferred the minus lens while 17 preferred the plus lens. Again, we tested whether perceived stereopsis was different between these two groups and this time we did find a significant result ( U=258.5 , p=.017 ) between the minus lens (mean = 0.67) and plus lens (mean = 0.26).

### Phase 2: Ratings of Specific Qualities

After the first phase of the experiment, observers were asked to view all paintings again while answering the specific questions about the perceived effect. Observers gave a Likert rating and verbal description. We will first present the rating data.

**Figure 4.**
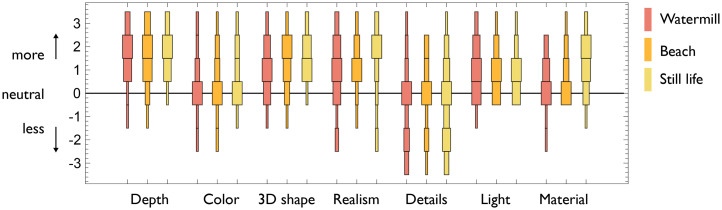
Results of Phase 2: Likert ratings per painting. The chart elements represent frequency data, thus serving is histograms per attribute and painting.

Per attribute, observers gave a Likert rating between 1 and 7 where 4 denoted no effect. For data analysis, the ratings were translated to [−3,3] . Distribution plots are shown in Figure 4. To test whether ratings were statistically different from neutral, we conducted Sign tests, of which the results are presented in Table 3. The relevant statistics for the Sign test are the number of positive ratings $\left(S^{+}\right)$ and number of nonzero ratings (n) . As can be seen, most of the attributes are significant in the positive direction, and rather stable across the three paintings. It should be noted that the statistical power of color, details, and material is relatively weak due to the high number of neutral ratings, and thus low n . The only attribute that did not reach statistical significance was details. Interestingly, those distributions appear bimodal, with modes at − 2 and 0, but also a substantial tail in the positive direction. Visually, it also clearly yields the largest rating range, which together suggests that ratings for details are rather idiosyncratic.

**Table 3.** Sign test results across the three paintings.

	Watermill				Beach				Still Life			
	Mean	$S^{+}$	n	p	Mean	$S^{+}$	n	p	Mean	$S^{+}$	n	p
Depth	1.61	34	36	<.001	1.53	32	33	<.001	1.71	35	35	<.001
Color	0.45	14	18	.031	0.45	15	19	.019	0.68	16	17	<.001
3D shape	1.03	26	28	<.001	1.29	30	31	<.001	1.55	34	34	<.001
Realism	0.92	25	29	<.001	1.16	29	33	<.001	1.13	27	34	.001
Details	−0.45	9	25	.230	0.03	12	21	.664	−0.42	11	28	.345
Light	1.03	25	26	<.001	1.11	26	26	<.001	1.13	26	26	<.001
Material	0.55	18	20	<.001	0.66	17	17	<.001	1.26	29	30	<.001

$S^{+}$ denotes number of positive signs, n denotes the number of nonzero signs.

As we had found a significant effect of lens polarity in Phase 1, we also tested whether the overall Likert ratings were higher for the minus lens than the plus lens, but did not find a similar difference ( ${\bar{x}}_{\min}=0.93$ , ${\bar{x}}_{\mathrm{plus}}=0.81$ , U=82591 , p=.22 ). Furthermore, instead of aggregated Likert ratings over all attributes to test the influence of lens polarity, we analyzed the details data separately. As the results in the previous paragraph suggested idiosyncrasy, we verified whether lens polarity affected the details rating. Indeed, details were judged higher for the minus lens than for the plus lens ( ${\bar{x}}_{\min}=0.0$ , ${\bar{x}}_{\mathrm{plus}}=-0.37$ , U=2003.5 , p=.02 ).

To distill information from users’ responses, we let Claude create clusters per attribute. We used all responses over all three paintings and used the prompt:

Below you will find responses from participants in a perception experiment about viewing paintings. They were asked if they saw any differences with respect to [attribute], when viewing the paintings with a certain device. The numbers repeat three times because they were looking at three different paintings, but you can combine these answers per respondent. Could you cluster these responses and list the respondent numbers for each cluster? Preferably no more than 3 clusters unless it’s really not possible otherwise. The responses are in Dutch, but please answer in English.

Below we report the clustering per attribute. Overall, the verbal data is well aligned with the quantitative Likert ratings.

#### Depth

Three clusters were described as significant ( N= 15), moderate ( N= 17), and minimal/unclear ( N= 6) depth perception. The significant group reported ‘‘distinct layers, clear separation between foreground and background” and ‘‘multiple depth planes.” Furthermore, it was reported that this was consistent over all paintings. The Moderate group observed ‘‘subtle depth effects, limited to specific areas or inconsistent across paintings.”

#### Color

The three clusters were named enhanced color/contrast ( N= 12), subtle or mixed ( N= 14) and minimal or no ( N= 11) with one respondent left out due to the lack of response. The enhanced color/contrast cluster was characterized by references to the liveliness (vivid, ‘‘levendig’’ in Dutch), specific colors increasing in prominence (especially blue and red) and increased contrast. The subtle of mixed cluster was characterized by having difficulty articulating precise differences or used phrases like ‘‘a little bit.” The minimal or no group hardly gave responses.

#### 3D Shape

The three clusters were named strong ( N= 14), moderate ( N= 13), and minimal or inconsistent ( N= 11). The strong cluster mentioned objects coming forward and appearing more rounded. The moderate group described the effects as subtle, and the minimal or inconsistent group reported no effects or gave unclear responses.

#### Realism

The three clusters were named enhanced realism through depth/3D effect ( N= 19), enhanced realism through visual changes ( N= 11, with one participant also present in the first cluster) and neutral or negative effect on realism ( N= 9). The enhanced realism through depth/3D effect was characterized by similar responses as the depth and 3D attributes (i.e., depth separation between foreground and background, more three-dimensionality). The enhanced realism through visual changes mentioned brush strokes becoming less visible, colors appeared more vibrant, reflective surfaces (like chrome and water) looked more realistic, blurriness sometimes made the painting seem more like a real scene and, finally, sharpness and contrast enhancing realism. The neutral or negative effect on realism cluster mentioned the effect being distracting, uncomfortable, ‘‘gimmicky’’ and artificial.

#### Details

The three clusters were named decreased detail ( N= 15), increased detail/contrast ( N= 7), mixed/neutral ( N= 8) and then there were unclassifiable participants ( N= 8). The decreased detail cluster was characterized by vagueness, less sharpness, blurriness, and focus difficulty. The increased detail/contrast cluster mentioned noticing specific details previously unseen, better contrast and sharpness and specific elements becoming more noticeable.

#### Light

The three clusters were named strong light enhancement ( N= 17), modest or mixed light ( N= 9) and minimal or no light difference ( N= 10) and two participants were not included. The strong light enhancement cluster was characterized by pronounced highlights, better contrast, more pronounced shadows, improved depth perception. The modest or mixed light cluster mentioned some light effects but not consistent, only in specific parts of the paintings and having contradictory responses across paintings.

#### Material

The three clusters were described as water, reflective and shiny objects ( N= 13), texture and detail ( N= 10), and little to no difference ( N= 15). The water, reflective and shiny objects cluster mentioned the water appearing more realistic or reflective, shiny materials (metal and glass) looking more authentic, glistening effects becoming more pronounced and furthermore there was specific mentioning of the waterfalls, ponds, silver kettles, and glass objects. The texture and detail cluster mentioned changes in detail (both enhanced and reduced), textural differences becoming more apparent and vagueness or softness affecting perception.

### Other Findings: Pollock

We reduced Phase 2 to three paintings and discontinued with the Pollock. Nevertheless, the combined results of Phase 1 with the 12 participants that did see Pollock in Phase 2 are interesting to report separately. In Phase 1, many participants ( N= 15) experienced the painting negatively with the lens: 11 participants commented about the unsharpness, four participants gave less specific negative comments. Six participants were positive but reported qualities unrelated to stereopsis, and seven did not report anything. A total of 10 participants experienced stereopsis (seven more depth, three more contrast and three sharper, some participants reported multiple qualities). From Phase 2, we have data of 12 participants. According to a similar Claude analysis that we performed in the general Phase 2 analysis, about half of the participants perceived a 3D effect. White and black splatters appeared at different depths, white highlights ‘‘popped out,” the painting appeared to have more body, and enhanced color perception was reported. During a debrief, various participants noted they could better organize elements in their mind or ”pull out separate elements.” Given the abstract nature of the painting, we believed it was interesting to share these insights, despite the limited data.

## Discussion

We tested for the first time whether a cylindrical lens in one eye could evoke stereopsis when looking at pictures. The experiment confirmed that naive participants spontaneously describe the effect in a similar manner as previously reported for monocular stereopsis (Vishwanath & Hibbard, 2013), which became even clearer when specific questions about stereoptic qualities were asked in Phase 2.

The advantage of introspective reports, where observers verbally share their visual experience, is that it is unbiased. The challenge is to analyze these qualitative accounts, which currently is facilitated by using generative AI models, such Claude (Anthropic, 2025). Although it is difficult to quantify the reliability of this novel analysis technique, Claude rating data correlated well with the experimenter ratings and the clustering was aligned with previous stereopsis studies. The combination of clustering and ratings suggests that the most prominent effect experienced by observers is depth, reported by approximately two out of three observers. Color and contrast were much less observed, and for material there was an equal amount of positive and negative responses. It should be noted that these are individual differences and do not cancel each other out. Furthermore, it is difficult to directly compare the results between the naive and (somewhat) informed viewing as the qualities differed but overall they seem very well aligned.

Despite Ames’ suggestion of using a plus lens, our own prior, informal experiences seemed to favor a minus lens. Hence we initially choose for a minus lens, but also tested for polarity preference. Furthermore, since the Ames’ Glass is an asymmetric optical device only applied to one eye, we additionally investigated whether there was a relation with eye dominance. Since lens polarity was not an initial independent variable (all participants used a minus lens initially) we could not measure the direct relation to perceived stereopsis. However, of the people who preferred the minus lens, the perceived stereopsis was higher than for the plus lens. Thus, there is a relation but not necessarily a causation between lens polarity and perceived stereopsis. A potential reason for a minus preference is that with accommodation it is partially (because cylindrical instead of spherical curvature) possible to refocus, which is not possible for a positive lens when looking at something relatively far away. Furthermore, the eye dominance investigation did not yield a certain pattern: we neither found a relation between lens position and perceived stereopsis, nor a preference for the dominant of nondominant eye. If there is truly no relation, which clearly cannot be confirmed as it is a null hypothesis, that would be good news for possible applications: designing Ames’ Glass spectacle frames would be more challenging when the effect substantially depends on eye dominance.

In Phase 2, we could probe perceived stereopsis in more detail by informing about the seven qualities. The first aspect to note is that the data is very similar for the three different paintings. The only two notable differences that can be observed in Figure 4 is that the Still Life seems to evoke higher realism and material ratings than Watermill and Beach. The purpose of 17th century Dutch Still Lifes was indeed to excel in realism and material rendering (Di Cicco, 2022). Although this does not explain stereopsis, it suggests that in order to experience the various qualities of stereopsis, the painting should afford amplifying these qualities. In hindsight, it could have been interesting to involve a dummy variable question to account for response bias. For example, we could have asked about the content instead of the form. Yet, it is difficult to predict what attribute would be totally independent of changes in pictorial form.

An interesting quality is sharpness in Phase 1 and the related details in Phase 2. While in Phase 1 a majority reported a degraded effect, a minority of five participants reported a positive effect. Experiencing less detail with wrongly prescribed glasses is clearly to be expected, but why are five participants reporting more detail? It could be that these participants happen to exactly need a horizontal cylindrical correction of − 1D, but that seems unlikely. The more likely explanation is that this is a side effect of stereopsis and perceiving something to be “real,” as reality itself is practically infinitely detailed. In Phase 2, the bimodality of the details rating as shown in Figure 4 indicates a similar idiosyncrasy: some participants experienced detail worsening while others experienced detail enhancement. This is supported by the analysis of the verbal responses where 15 participant experienced “Decreased Detail” and seven participants experienced “Increased Detail.” It has been reported by Barry (2009), who described binocular stereoscopic experience after regaining binocular stereo vision, that sharp edges seemed to emerge everywhere. Also Vishwanath and Hibbard (2013) report a sense of increased sharpness and detail. Like in our study, it was not the most dominant effect, but it was present. Especially for the Ames’ Glass, which optically deteriorates the visual signal, it is surprising that some subjective experiences concern a sharpness increase instead of decrease.

One of the qualities that did not emerge from Phase 1 was miniaturization (closer and smaller observations), which was one of the key qualities proving the stereopsis theory of Vishwanath (2014). A possible explanation for this absence could be that participants were viewing the screen from 2.8 m, which is outside the range for accommodation to work as a depth cue (Fisher & Ciuffreda, 1988). Thus, accommodation cannot be re-attributed in a similar fashion as reported by Vishwanath and Hibbard (2013). Aside from the absence of miniaturization observations, our qualitative description of stereopsis largely agrees with monocular aperture viewing (Vishwanath & Hibbard, 2013): depth, 3D shape, and realism dominated the impression, with smaller but significant roles for light and material. The only difference concerns reports of changes in color, which was less prominent in our experiment. Above, when discussing the higher ratings of material in the Still Life painting, we suggested that paintings should afford the enhancement of stereoptic qualities, otherwise there is nothing to enhance. This could be the explanation for the relatively weak color enhancement in this present study: perhaps the color distribution was too small to be enhanced.

In a taxonomy of pictorial spacecrafts, as presented in Table 1, the Ames’ Glass takes an interesting position. What all optical aids have in common is that they perturb the nonpictorial cues signifying its physical flatness, but they do so in different ways. The Zograscope, Graphoscope, Perspectoscope, and Shomescope all make use of a convex lens or concave mirror which serves as local prisms that decrease (con)vergence towards parallel, that is, projected far away. The accommodation is likewise altered such that the image seems far (Koenderink, 2012). For the lens based devices (Zograscope and Graphoscope), chromatic aberrations can potentially elicit chromostereopsis. The latter is a byproduct but can elicit surprising effects (it would only work effectively for pictures respecting color-depth relations dictated by the laws of refraction). The Verant and the Plastoscope share that they are both monocular, but differ in other respects. The Verant also uses a lens (the Verant *is* a lens), and is specifically designed to cancel monocular motion parallax (Koenderink, 2012), while the Plastoscope is merely an monocular aperture, thus comparable with Vishwanath and Hibbard (2013). Although vergence (Linton, 2020) and accommodation (Mon-Williams & Tresilian, 2000) do not seem effective depth cues by themselves, perturbing them apparently affects stereopsis. Perhaps moving the eyes over a surface does give vergence and accommodation a stronger depth cue than in isolated sampling conditions such as the experiments cited above.

The Plastoscope, Synopter, and Ames’ Glass differ in many respects, but they share the possibility of use at variable distances: unlike other pictorial space crafts, they can be used to view pictures in a gallery instead of from a fixed, nearby position. What makes the Ames’ Glass unique is the way it modulates the binocular disparities while not affecting vergence, at least not in the way the synopter enforces parallel vergence. Despite these differences, the evoked stereopsis shares many similarities with previously found monocular aperture viewing.

Stereopsis, whether evoked by pictorial space crafts as reported here, or by different viewing methods such as shifting accommodation focus (Vishwanath, 2016), continues to surprise both observers and researchers. Recently, Papathomas and Rogers (2025) self reported on a case of monocular stereopsis after third-nerve palsy (paralysis) in one eye. The author/observer reported the effect was stronger than synoptic or monocular viewing. However, it could well be that different methods or devices affect observers idiosyncratically. We have observed some idiosyncrasy within sharpness and details, and it may well arise over different pictorial space crafts. Only one study compared two devices (Wijntjes, 2017) but unfortunately that was, as discussed previously, probably a nonideal version of the Ames’ Glass as it had a strong spherical instead of weak cylindrical curvature. A study comparing all these ways of experiencing stereopsis would be interesting but will also pose challenges. The effects sometimes appear to come and go and change from day to day. Also, there may be hysteresis or after effects which are also unexplored.

In sum, we contributed valuable insights into a peculiar pictorial space craft: the Ames’ Glass. How stereoptic effects rely on the type of pictorial space craft, how stable stereopsis is, and what individual traits determine individual differences, are all intriguing directions for future research.

## Notes

1. In fact, they used a graphoscope but the title refers to the zograscope. Both devices make use of a large lens, but for the zograscope an extra mirror facilitates viewing pictures that lie flat on a table.
